# Repeated High Intensity Bouts with Long Recovery: Are Bicarbonate or Carbohydrate Supplements an Option?

**DOI:** 10.1155/2014/145747

**Published:** 2014-11-10

**Authors:** Thomas Stöggl, Rafael Torres-Peralta, Ebru Cetin, Masaru Nagasaki

**Affiliations:** ^1^Department of Sport Science and Kinesiology, University of Salzburg, Schlossallee 49, 5400 Hallein/Rif, Austria; ^2^Swedish Winter Sports Research Centre, Department of Health Sciences, Mid-Sweden University, Studentplan 4, 83140 Östersund, Sweden; ^3^Department of Physical Education, University of Las Palmas de Gran Canaria, C/Juan de Quesada, No. 30 35001 Las Palmas de Gran Canaria, Spain; ^4^Gazi University School of Physical Education and Sports, Gazi University, Teknikokullar, 06500 Ankara, Turkey; ^5^Department of Health Science, Faculty of Psychological and Physical Science, Aichi Gakuin University, 12 Araike, Iwasaki-cho, Nisshin, Aichi 470-0915, Japan

## Abstract

The effects of varying recovery modes and the influence of preexercise sodium bicarbonate and carbohydrate ingestion on repeated high intensity performance, acid-base response, and recovery were analyzed in 12 well-trained males. They completed three repeated high intensity running bouts to exhaustion with intervening recovery periods of 25 min under the following conditions: sodium bicarbonate, active recovery (BIC); carbohydrate ingestion, active recovery (CHO); placebo ingestion, active recovery (ACTIVE); placebo ingestion, passive recovery (PASSIVE). Blood lactate (BLa), blood gases, heart rate, and time to exhaustion were collected. The three high intensity bouts had a duration of 138 ± 9, 124 ± 6, and 121 ± 6 s demonstrating a decrease from bout 1 to bout 3. Supplementation strategy had no effect on performance in the first bout, even with differences in pH and bicarbonate (HCO_3_^−^). Repeated sprint performance was not affected by supplementation strategy when compared to ACTIVE, while PASSIVE resulted in a more pronounced decrease in performance compared with all other interventions. BIC led to greater BLa, pH, and HCO_3_^−^ values compared with all other interventions, while for PASSIVE the opposite was found. BLa recovery was lowest in PASSIVE; recovery in pH, and HCO_3_^−^ was lower in PASSIVE and higher in BIC.

## 1. Background

Many sports (e.g., team sports such as soccer, rugby, and ice hockey; combat sports like judo; endurance sports like swimming, cycling, and running) often require short duration maximal or near-maximal efforts to be regularly repeated over an extended period of time [1]. In particular, the cross-country skiing sprint competitions have four maximal bouts with durations of 2–5 minutes within 2-3 hrs (including the qualification bout). In the finals, the skiers have to reproduce maximal performance over 3 bouts with only 10–25 min rest in between [2]. Therefore, understanding the physiological load and the recovery kinetics of various physiological parameters (e.g., blood pH, blood lactate (BLa), etc.) and their effects on subsequent repeated performance is essential.

During a sprint as short as 30 s, muscle glycogen stores are supposed to be partially consumed and, at the end of long duration training (60–90 min) that involves high, moderate, and low bouts of exercise, muscle glycogen will be dramatically reduced or even depleted [3]. In a simulated alpine skiing slalom training with 16 times 45 s runs (one run every 20 min), it was demonstrated that after the fourth run the intramuscular glycogen store was reduced by 30% and after 16 runs by 71%, especially in type 1 fibers [4]. Stöggl et al. [2] demonstrated that, during a cross-country skiing sprint, performance and peak BLa decreased from heat to heat and the magnitude of BLa was positively related with sprint performance. Based on their results, both Stöggl et al. [2] and Vogt et al. [4] recommended carbohydrate supplementation prior to, during, or after the race/training.

Several studies showed that ergogenic supplements positively affected sprint performance, as well as postperformance recovery (e.g., [5–7]). Carbohydrate (CHO) based supplements are probably the most common. There is an ongoing debate among coaches and athletes concerning whether CHO supplementation during repeated high intensity sprint competitions is a solution (e.g., among cross-country skiing sprint skiers). However, research on intermittent or high intensity and repeated sprint ability together with CHO loading or supplementation is sparse [8, 9]. Supplementation with CHO prior to or during a time trial resulted in performance improvements due to an increase in CHO oxidation and maintained plasma glucose levels [10].

Additionally, the use of sodium bicarbonate as one of the major buffering agents used to diminish the supposed negative effects of acidosis is common [11]. Recent research has shown that ingesting sodium bicarbonate may enhance aspects of sprint performance such as power output, total anaerobic work, and delaying fatigue [11, 12], though results are not consistent. A recent meta-analysis revealed that ingestion of bicarbonate improves mean power by 1.7% (±2.0%) in high intensity races of short duration [13]. Lavender and Bird [14] and Bishop et al. [15] concluded that sodium bicarbonate supplementation improves power output levels in repeated short duration sprints (e.g., 10 times 10 s sprints with 50 s recovery, or 5 times 6 s all-out sprints every 30 s). Bishop et al. [15] suggested that the improved performance was a result of the greater extracellular buffer concentration by sodium bicarbonate ingestion increasing H^+^ efflux from the muscles into the blood and an increased anaerobic energy contribution. Research on repeated high intensity bouts of longer duration and with longer recovery in between is lacking.

Finally, the role of the recovery mode during repeated high intensity activity is also a topic of debate. Active recovery (pedaling at 20% VO_2max⁡_) has been shown to facilitate performance (4 times 110% peak performance to exhaustion with 5 min recovery in between bouts) compared to passive recovery [16], while the opposite was found in intermittent exercise of 15 s high intensity versus 15 s recovery (40% VO_2max⁡_ during active recovery) [17] and with no differences between 3 times 110% peak power output to exhaustion with 12 min breaks (20% of maximal workload during active recovery) in between [18]. Furthermore, passive recovery is expected to favor PCr resynthesis during shorter recovery periods between bouts, for example, up to 2 min [7].

Therefore, the aim of the current study was to analyze the effects of passive versus active recovery and how carbohydrate and sodium bicarbonate supplements influence performance and recovery during three bouts of high intensity exercise with recovery duration of 25 minutes in between. The specific hypotheses were that (a) active recovery facilitates repeated performance, (b) acute sodium bicarbonate ingestion leads to performance enhancement in each high intensity bout, and (c) acute carbohydrate supplementation delays fatigue across all three high intensity bouts.

## 2. Methods

### 2.1. Subjects

Twelve endurance-trained males (mean ± SD: age = 32.8 ± 3.8 yrs, body height = 1.78 ± 0.06 m, body weight = 74 ± 6 kg, and VO_2max⁡_ = 66.4 ± 5.2 mL*·*min^−1^ *·*kg^−1^) with backgrounds in running and cross-country skiing volunteered to participate in this study.

### 2.2. Ethics Statement

Subjects were informed about the test procedures and possible risks prior to giving their written informed consent to participate. The research techniques and experimental protocol were preapproved by the Local Ethics Committee of the University of Salzburg, and the study was performed in accordance with the Declaration of Helsinki.

### 2.3. Overall Design of the Study

Participants reported to the laboratory at the same time of the day on four separate test days with a minimum of 5 days, but no more than 8 days between trials. For all participants, all tests were performed within 23 ± 3 days. Participants were advised not to perform strenuous exercise within 72 hrs prior to each trial and were asked to control, record, and duplicate food intake 24 hrs prior to each trial (data collected and reviewed, but not presented in this paper). Each test day included a standardized warm-up followed by three all-out sprint bouts running on a treadmill with fixed breaks of 25 min in between bouts (comparable with the competition mode in sprint cross-country skiing). Each test had a total duration of approximately 90 min. On each test occasion the following supplementation and recovery strategies were randomized (counterbalanced using the Latin least squares design) in a double-blind manner across the participants: (a) placebo drink with no activity between sprint bouts (PASSIVE); (b) placebo drink and low intensity running between sprint bouts (ACTIVE); (c) carbohydrate mix and low intensity running between sprint bouts (CHO); and (d) bicarbonate drink and low intensity running between sprint bouts (BIC).

### 2.4. Supplementation

The BIC drink was prepared according to McNaughton [19] using 300 mg/kg (body mass) bicarbonate and 2 cl artificial sweetener (saccharin) dissolved in 6 mL/kg (body mass) water. The CHO mixture was an isotonic 5.5% solution, which consisted of 1.7% glucose; 1.1% fructose; 0.6% maltose; 1.9% (higher) saccharide and electrolytes (sodium: 61 mg/100 mL and potassium: 10 mg/100 mL) [20] with total CHO amount of 300 mg/kg (body mass). The placebo drink (PASSIVE and ACTIVE group) consisted of artificial sweetener (saccharin) and sodium chloride. For CHO, PASSIVE, and ACTIVE the participants ingested 6 mL/kg (body mass) of the respective solution at arrival and the same amount throughout the experiment.

### 2.5. Experimental Protocol

Participants reported to the laboratory 100 min before test start with no food intake 3 hrs prior to the test start; they were instructed to be refrained from caffeine and alcohol and be well-hydrated. Ten min after arrival, two blood samples from the earlobe were taken to determine resting values in blood lactate (BLa) and blood gases. Directly afterwards (90 min prior to the start of the exercise protocol) the participants drank, according to the randomized order, one of the three supplements [placebo (PASSIVE, ACTIVE), CHO, and BIC] within a 10 min period. In addition, ten minutes prior to warm-up, as well as within the breaks between the single bouts, participants drank 2 mL/kg of CHO or placebo (for PASSIVE, ACTIVE, and BIC). Another two blood samples for BLa and blood gases were collected 3 minutes prior to start of the warm-up. The CHO was administered throughout the protocol in order to possibly enhance CHO oxidation and maintain plasma glucose levels [10]. BIC supplementation was switched to the placebo drink during the exercise, in order to prevent possible gastrointestinal problems. The test started with a standardized warm-up protocol of 15 min, with 5 min at 8 km/h at 1.5% grade, 5 min at 8 km/h at 5% grade, and 3 times 30 s acceleration runs up to 16, 18, and 19 km/h at 5% grade with 1 min at 8 km/h in between the sprint runs. The warm-up was finished with 1.5 min at 8 km/h at 5% grade. In the first minute, after warm-up, two blood samples were taken: a blood sample for the determination of BLa and one for the determination of blood gases. After a rest of 3 min, the first high intensity bout started with a fixed treadmill inclination of 5% and a treadmill speed of 19 km/h. Participants were asked to run as long as possible at this treadmill speed and were maximally encouraged by the test team. The time to exhaustion was taken as the performance parameter for each sprint bout. The participants had no knowledge of the length of time they were actually running. Even though time trial tests were shown to have higher validity than a time to exhaustion test [21], we decided to use this test concept based on (1) high internal validity by maximal standardization of the running test on an indoor treadmill and (2) prevention of different pacing strategies during each of the four trials. Furthermore, the participants were familiar with this test procedure based on a reliability study prior to this experiment (*n* = 15 including the same subjects of this study, intraclass coefficient of 0.95; *P* < 0.001 for maximal test duration).

**Figure 1:**
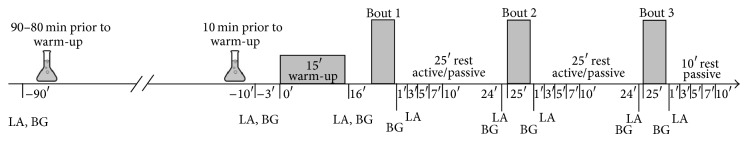
Illustration of the general design of the study.

After each bout, the participants had a 25 min break consisting of 5 min rest followed by 17 min of either sitting on a chair (PASSIVE) or low intensity running [~65–70% peak heart rate (HR)] on the treadmill at 6 km/h and a 1.5% grade (ACTIVE, CHO, and BIC) and another 3 min passive rest prior to the second sprint bout. The same procedure was repeated for the third sprint bout. Following each sprint bout blood samples for BLa were taken in the 3rd, 5th, 7th, 10th, and 25th min and for blood gases in the 1st and 24th min. An overview on the entire protocol is illustrated in Figure 1. All participants were secured with a safety harness suspended from the ceiling and attached to an emergency break.

### 2.6. Instruments

The participants' HR was recorded throughout all tests telemetrically (Suunto t6, Helsinki, Finland) at 2 s intervals. The peak HR of each bout was used for further statistical analysis. For BLa a 20 *µ*L and for blood gases a 150 *µ*L blood sample from the hyperaemized right earlobe were collected into a capillary tube (Eppendorf AG, Hamburg, Germany). The BLa samples were immediately analyzed amperometric-enzymatically (Biosen 5140, EKF-Diagnostic GmbH, Magdeburg, Germany). The lactate sensor was calibrated before each test and checked using a lactate standard sample of 12 mmol/L. Results within a range of ±0.1 mmol*·*L^−1^ were accepted. Blood gases were analyzed using the Cobas b 221 system (Roche Diagnostics GmbH, Mannheim, Germany). The system automatically performs a system calibration every 24 hrs and a 1-p calibration every 30 minutes.

### 2.7. Statistical Analysis

All data were checked for normality using Shapiro-Wilk's test to exhibit a Gaussian distribution, and the values are presented as mean ± SD. For determination of global differences between measured physiological parameters (BLa, pH, HCO_3_^−^, base excess (BE), PO_2_,  and PCO_2_) across the entire experiment (all three bouts including the postrecovery values) a 4 × 5 repeated-measures ANOVA (4 interventions × 5 time points) was applied. For the variables sprint bout time and the peak values of the measured physiological parameters during or after sprint a 4 × 3 repeated-measures ANOVA (4 interventions × 3 sprint bouts) was conducted. For determination of differences within the single sprint bouts and the single recovery values an ANOVA with repeated measures was used. In all cases a Bonferroni post hoc analysis was applied. Correlations of performance in the first bout to the peak values and recovery values in BLa and pH Pearson's product moment correlation coefficients were calculated. Level of significance was set at *α* < 0.05.

## 3. Results

Upon arrival at the laboratory, there were no differences indicated for pH, lactate, HCO_3_^−^, BE, PO_2_, or PCO_2_ between the four interventions (Table 1). Some participants did report experiencing minor bloating and/or gastrointestinal stress following the BIC (*n* = 2) and CHO (*n* = 1) within the 90 min after ingestion time period. However, these side effects subsided by the time the warm-up started.

**Figure 2:**
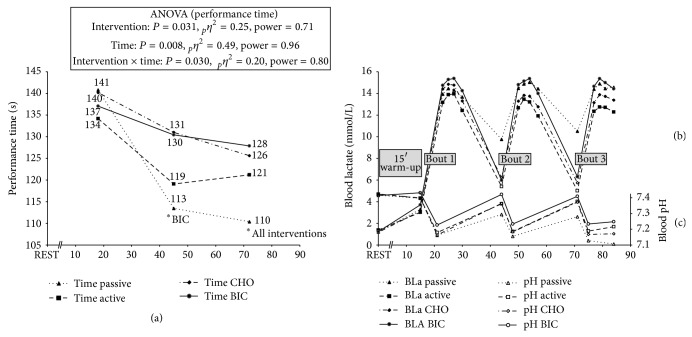
Development of running performance (a), blood lactate (b), and pH (c) across the entire experiment including resting values, post-warm-up values, and postbout values. PASSIVE: passive recovery with placebo; ACTIVE: active recovery with placebo; CHO: active recovery with carbohydrate ingestion; BIC: active recovery with sodium bicarbonate ingestion. ^*^Significant difference to specified intervention.

High intensity running performance reflected by the sprint bout duration demonstrated a main effect for intervention (mean of all three bouts) (PASSIVE: 122 ± 6 s; ACTIVE: 125 ± 5 s; CHO: 132 ± 8 s; BIC: 132 ± 9 s; *P* = 0.031) with no difference at bout 1, but lower performance in the PASSIVE group at bout 2 compared with BIC (*P* = 0.048) and at bout 3 compared with all other groups (*P* = 0.009 to 0.012). This was also reflected by an interaction effect towards greater decreases in running performance with PASSIVE compared with BIC from bout 1 to bout 2 (*P* = 0.05) and when compared with BIC and CHO from bout 1 to bout 3 (PASSIVE: −30.5 ± 17.9 s; CHO: −14.5 ± 13.2 s; BIC −9.2 ± 16.9 s; *P* = 0.029). There was a main effect for factor time (*P* = 0.008) demonstrating a tendency towards a decrease in performance (−10%) from bout 1 to bout 2 (138 ± 9 s versus 124 ± 6 s, *P* = 0.066) and when comparing bout 1 with bout 3 (−12%: 138 ± 9 s versus 121 ± 6 s, *P* = 0.007) (Figure 2(a)).

Peak HR within each bout demonstrated a main effect for intervention (PASSIVE: 181 ± 2 bpm; ACTIVE: 185 ± 2 bpm; CHO: 184 ± 2 bpm; BIC: 186 ± 2 bpm; *P* = 0.009) with higher values in BIC compared with PASSIVE (*P* = 0.026) and a tendency towards higher values in ACTIVE compared with PASSIVE (*P* = 0.062). When analyzing each bout separately, there was no difference between interventions at bout 1, while at both bout 2 (*P* = 0.015) and bout 3 (*P* < 0.001) PASSIVE was lower in peak heart rate compared with all other interventions. A main effect was also found for factor time with a reduction in peak HR across bouts (bout 1: 189 ± 2 bpm; bout 2: 182 ± 2 bpm; bout 3: 181 ± 2 bpm; *P* < 0.001). An interaction of time × intervention was found (*P* < 0.001), with greater decreases from bout 1 to bout 2 and bout 1 to bout 3 in PASSIVE compared with all other interventions.

### 3.1. Peak Values after Sprint Bout

Physiological parameters across the entire experiment for all four interventions are illustrated in Figure 2 and Table 1. Peak BLa values demonstrated a main effect for intervention (*P* = 0.002), leading to greater BLa values in the BIC group compared with CHO and ACTIVE, with no change across time. An interaction effect between time and intervention (*P* = 0.045) was found, demonstrating a decrease in BLa for ACTIVE and CHO compared with stable values in BIC and PASSIVE. Postbout blood pH values demonstrated main effects for intervention (*P* < 0.001) with no time or interaction effect with higher values in BIC compared with all other interventions. When examining the postbout HCO_3_^−^ and BE values there was a main effect for intervention and time (all *P* < 0.001) with higher values for BIC compared with all other interventions and a decrease in the values from bout to bout. Postbout PO_2_ values reflected no differences between interventions and no change across sprint bouts. PCO_2_ was greater in BIC compared with all other interventions (*P* = 0.016 to 0.050) with a reduction from bout 1 to bout 2 (*P* = 0.014) and bout 1 to bout 3 (*P* = 0.006).

### 3.2. Recovery Values after 25 min Recovery

Relative to the BLa value after the 25 min recovery, there was a main effect for intervention (*P* < 0.001) with no time effect, which demonstrated higher BLa values in PASSIVE compared with all other interventions. In regard to BLa recovery (delta changes from the peak values to the values at the end of the 25 min recovery phase), a main effect for intervention was found (PASSIVE: −4.8 ± 0.3 mmol/L; ACTIVE: −8.7 ± 0.4 mmol/L; CHO: −8.6 ± 0.2 mmol/L; BIC: −9.5 ± 0.3 mmol/L; *P* < 0.001) with lowest reduction in BLa in PASSIVE compared with all other interventions and a trend towards greater reduction in BIC compared with CHO (*P* = 0.059). Furthermore, CHO had lower recovery compared with BIC (*P* = 0.037). Blood pH, HCO_3_^−^, and BE values after the 25 min recovery demonstrated a main effect for intervention (all *P* < 0.001) with no time or interaction effect with lower values in PASSIVE and higher values in BIC, respectively, compared with all other interventions. There was no difference in PO_2_ values after 25 min recovery, while for PCO_2_ PASSIVE was lower compared with all other interventions (*P* = 0.010 to 0.050).

### 3.3. Correlations

Performance in the first bout (test duration) was correlated with peak BLa, peak pH, and the recovery values after 25 min for BLa and pH for PASSIVE (*r* = 0.74, −0.88, 0.88, and −0.94; all *P* < 0.001), ACTIVE (*r* = 0.73, −0.82, 0.73, and −0.69; all *P* < 0.05), and CHO (*r* = 0.65, −0.72, 0.54, and−0.63; all *P* < 0.05). In the BIC situation only peak pH and pH after 25 min recovery were related with performance in the first bout (*r* = −0.63, *P* < 0.05; *r* = −0.80, *P* < 0.01). No such correlation was found for peak and recovery BLa (*r* = 0.41, 0.53; both *P* > 0.05).

## 4. Discussion

The main findings of the current study were that (a) the supplementation strategy had no effect on the performance in the first sprint bout, even though marked differences in pH, BE, and HCO_3_^−^ were found, particularly in the BIC intervention; (b) repeated sprint bout performance was not affected by supplementation strategy when compared to the placebo active condition (ACTIVE), while placebo with passive rest between the bouts (PASSIVE) resulted in a more pronounced decrease in performance; (c) BIC led to greater BLa, pH, HCO_3_^−^, and BE values across the entire experiment compared with all other interventions, while for PASSIVE the opposite was found; (d) BLa values did not change across repeated sprint bouts, though peak HR and performance dropped; (e) though globally not changed, BLa remained constant in BIC and PASSIVE while it decreased in CHO and ACTIVE across sprint bouts; (f) BLa recovery during the 25 min recovery periods was lowest in PASSIVE compared with all other interventions; recovery in pH, BE, and HCO_3_^−^ was lower in PASSIVE and higher in BIC compared with all other interventions; and (g) performance in the first sprint bout was related to higher peak BLa and lower pH values after exercise and after the 25 min recovery within all interventions except for BIC where no correlations were found.

### 4.1. Passive versus Active Recovery

Motivated by the contrasting results of previous research on the effects of active versus passive recovery, the current study supports that active recovery during breaks of longer duration (i.e., 25 min) is a favorable strategy compared with passive recovery during repeated high intensity bouts. Active recovery (pedaling at 20% VO_2max⁡_) has been shown to facilitate performance compared to passive recovery in a protocol with 4 times at 110% of peak performance to exhaustion and 5 min recovery in between. This was based on an increase in aerobic energy yield and a higher fractional contribution of aerobic metabolism to total energy turnover [16]. Therefore, active recovery may have favored an increased aerobic contribution during the subsequent high intensity bouts and may have promoted an increase in muscle blood flow, O_2_ delivery, and a correspondent increase in intracellular pH for all active interventions [22, 23]. On the contrary, in another study [17] during intermittent exercise of 15 s high intensity with 15 s recovery until exhaustion was reached, the passive rest led to increased time to exhaustion compared with active rest using intensity of 40% VO_2max⁡_ based on lower muscle oxygenation levels during active than during passive recovery. Furthermore, despite comparable responses in acid-base status as in the current study, performance was unaffected by 12 min active versus passive recovery between 3 high intensity (110% peak power output) bouts to exhaustion in the study of Siegler et al. [18]. Another explanation for the reduced performance in the PASSIVE situation in the current study might be a decreased metabolic rate and/or priming of the neuromuscular and muscle-tendon system prior to the high intensity bouts.

The low active recovery intensity used in this study was an attempt to increase muscle blood flow while not interrupting PCr resynthesis, since those stores are crucial for subsequent efforts [24, 25]. When considering that the half time recovery for PCr is rarely superior to 90 s during a passive recovery [26], we expected a more than optimum reload of PCr in the current study for both active and passive interventions. Thus, we may rule out its influence on performance in our bouts without using invasive techniques. Accepting passive recovery to be favored towards PCr resynthesis in short recovery periods between bouts (e.g., up to 2 min [7]) is probably not imperative when using a much longer 25 min rest period as in the current study. Therefore, together with the findings of Dorado et al. [16], the current study supports that active recovery seems to lead to superior performance outcome in repeated high intensity bouts when the recovery period is at least 5 min. Further research is warranted concerning the detection of the actual turning point when active recovery outperforms passive recovery and the type of exercise and intensity used during active recovery.

### 4.2. Effects on Performance

Finding no performance differences between the CHO, BIC, and placebo intervention (ACTIVE), in contrast to other studies with shorter bout durations and recovery periods (e.g., [5–7, 11, 12]), we conclude that for repeated high intensity performance to exhaustion (bout duration ~2 min) with long recoveries (25 min) the length and activity mode of the recovery period may be a more crucial variable than the acid-base status or possibly enhanced glucose availability based on the CHO supplementation. This idea was previously proposed by Siegler et al. [7]. In order for bicarbonate supplementation to be effective, the metabolic stress must be enough to decrease glycolytic activity and diminish PCr resynthesis and Ca^2+^ resequestering in the cell so as to grant a decline in the force production. Furthermore, it would be necessary to maintain this stress situation until the next bout in order to possibly create a significant difference in performance. Though differences in pH for different active recovery strategies before the second and third bouts were found in the current study, repeated performance was not different between interventions with active recovery.

Concerning CHO supplementation, neither performance enhancement in the first bout nor increased fatigue resistance across all three bouts can be confirmed. Therefore, in agreement with earlier studies, CHO supplementation or loading seems to have no effect on sprint performance and/or high intensity exercise up to about 30 min compared with normal diets [27]. To our knowledge, there is only one study demonstrating that a chronic application of carbohydrates over five days resulted in an increase in high intensity performance (e.g., repeated countermovement jump performance with 10 jumps over 60 s) compared with a placebo [28]. The current study is in line with the findings of Jenkins et al. [3, 29], who demonstrated that there is no improvement in performance while in a carbohydrate loaded state and when ingesting glucose prior to the exercise during five 60 s all-out cycling bouts separated by 5 min of passive recovery compared with a normal carbohydrate diet. The authors questioned the ergogenic potential of consuming glucose before supramaximal exercise [3]. Also Robinson et al. [30] found no difference between the ingestion of carbohydrates versus placebo during high intensity running (intensity at 100% VO_2max⁡_) over a duration comparable to that of the current study. Therefore, the speculation of Stöggl et al. [2] that CHO supplementation might enhance performance in a cross-country skiing sprint competition cannot be supported based on the current findings. However, the finding that peak BLa decreased from bout 1 to bout 2 and that the magnitude of BLa values across the entire simulation was positively related to cross-country skiing sprint performance is in line with the findings of the current study where, in all active rest interventions except BIC, BLa decreased from bout 1 to bout 2 and also, in all but the BIC intervention, peak BLa was related to sprint performance. This result might indicate that, especially with BIC, participants show different responses that might have diluted the group outcome. Finally, the ergogenic effect of BLa as proposed by others [31–33] was not noted in this experiment, as PASSIVE and BIC demonstrated a higher prebout BLa but, respectively, lower and similar performance compared with the other interventions.

## 5. Limitations

One limitation of the current study might be seen in the collection of arterialized instead of arterial blood samples for determination of blood gases. In a study of Sauty et al. [34] it was demonstrated that the correlation coefficients between arterial and arterialized blood samples for PO_2_ and PCO_2_ were high with 0.928 and 0.957 (both *P* < 0.001). However, arterialized earlobe PO_2_ was lower than arterial PO_2_ in most cases, and the difference increased as arterial PO_2_ increased. Therefore, the absolute values of PO_2_ in the current study might underestimate the true PO_2_ values. However, based on the repeated measured design this fact might not affect the basic outcome.

## 6. Conclusion

In summary, the findings of the current study show that when successive high intensity maximal exhaustion bouts are separated by 25 min of recovery, an active recovery coupled with a preexercise metabolic alkalosis or carbohydrate supplementation would not lead to detectable improvements in performance as assessed by time to fatigue at high speed treadmill running, regardless of sustaining blood buffering capacity. In spite of a higher pH than the placebo condition in the case of the bicarbonate supplementation intervention and supposed higher glucose availability in carbohydrates supplementation, we found no significant difference in performance. Only the application of active recovery led to enhanced performance and attenuated fatigue when compared with the passive recovery intervention. Future studies are needed to gain deeper insight into the relationship between exercise intensities and durations with recovery modes and recovery intensities and supplementation after repeated high intensity activity in order to improve performance or delay fatigue.

## Figures and Tables

**Table 1:** Blood lactate, blood gases, pH, and base excess (BE) associated with four interventions at rest, pre-warm-up, post-warm-up, and before and after the three high intensity bouts (mean ± SD).

		Rest	Before warm-up	Warm-up	After bout 1	Before bout 2	After bout 2	Before bout 3	After bout 3
Blood lactate (mmol/L)	PASSIVE	1.2 ± 0.3	1.3 ± 0.2	3.3 ± 1.6	14.8 ± 2.0	9.8 ± 2.5^*^	15.2 ± 2.2	10.5 ± 2.7^*^	14.4 ± 2.5
	ACTIVE	1.4 ± 0.6	1.3 ± 0.3	3.0 ± 1.2	14.2 ± 2.8	5.4 ± 2.4	13.6 ± 1.9	5.0 ± 1.5	13.0 ± 1.8
	CHO	1.2 ± 0.2	1.3 ± 0.2	3.1 ± 1.4	15.0 ± 2.0	6.2 ± 2.2	14.1 ± 2.3	5.8 ± 1.8	14.0 ± 2.0
	BIC	1.2 ± 0.4	1.4 ± 0.5	3.7 ± 1.6	15.6 ± 2.4	6.0 ± 1.5	15.6 ± 2.1^†^	6.3.5 ± 1.6^‡^	15.5 ± 1.8^†‡^

pH	PASSIVE	7.41 ± 0.02	7.41 ± 0.01	7.41 ± 0.02	7.16 ± 0.04	7.29 ± 0.05^*^	7.16 ± 0.04	7.28 ± 0.06^*^	7.15 ± 0.06
	ACTIVE	7.43 ± 0.02	7.43 ± 0.03	7.41 ± 0.03	7.18 ± 0.05	7.39 ± 0.04	7.19 ± 0.05	7.39 ± 0.05	7.19 ± 0.04
	CHO	7.43 ± 0.02	7.43 ± 0.01	7.42 ± 0.02	7.16 ± 0.07	7.36 ± 0.05	7.19 ± 0.06	7.36 ± 0.06	7.17 ± 0.05
	BIC	7.41 ± 0.01	7.47 ± 0.02^*^	7.44 ± 0.01^‡$^	7.24 ± 0.06^†^	7.43 ± 0.04^†^	7.22 ± 0.06^*^	7.39 ± 0.05^†^	7.22 ± 0.04^*^

HCO_3_^−^ (mmol/L)	PASSIVE	24.2 ± 1.3	24.0 ± 1.1	21.6 ± 1.7	12.0 ± 2.0	13.5 ± 3.0^*^	10.1 ± 1.9^*^	12.4 ± 2.0^*^	9.4 ± 2.3
	ACTIVE	24.2 ± 1.7	24.4 ± 1.6	21.9 ± 1.7	13.0 ± 2.4	18.1 ± 3.3	11.4 ± 2.3	18.4 ± 2.1	11.8 ± 1.5
	CHO	24.2 ± 1.2	24.1 ± 1.5	21.6 ± 2.2	12.2 ± 1.9	17.8 ± 2.9	11.9 ± 1.9	18.4 ± 2.5	11.5 ± 2.3
	BIC	24.5 ± 1.4	24.3 ± 1.2	25.1 ± 2.7^*^	15.4 ± 2.9^†$^	21.2 ± 1.8^†$^	13.9 ± 2.0^*^	21.1 ± 2.8^†$^	13.5 ± 2.6^$^

BE (meq/L)	PASSIVE	−1.7 ± 1.2	−1.5 ± 1.2	−4.4 ± 1.8	−16.8 ± 2.2	−13.4 ± 3.6^*^	−18.6 ± 2.7^*^	−14.7 ± 2.6^*^	−19.7 ± 3.1
	ACTIVE	−1.6 ± 1.4	−1.6 ± 1.5	−4.1 ± 1.8	−15.5 ± 3.0	−8.2 ± 3.8	−17.0 ± 2.8	−7.7 ± 2.3	−16.5 ± 2.0
	CHO	−1.5 ± 1.3	−1.6 ± 1.2	−4.3 ± 2.3	−16.5 ± 2.4	−8.4 ± 3.3	−16.6 ± 2.6	−7.7 ± 2.8	−17.2 ± 3.0
	BIC	−1.4 ± 1.3	−1.1 ± 1.1	−0.6 ± 2.7^*^	−12.6 ± 3.3^†$^	−4.3 ± 2.2^†$^	−13.9 ± 2.7^*^	−3.9 ± 2.1^†$^	−14.3 ± 2.8^†$^

PO_2_ (mmHg)	PASSIVE	78.3 ± 5.7	78.5 ± 5.9	82.6 ± 4.4	93.2 ± 5.9	83.6 ± 7.9	95.8 ± 7.2	84.9 ± 8.5	98.2 ± 7.6
	ACTIVE	81.4 ± 9.3	80.1 ± 8.3	82.0 ± 4.7	91.7 ± 9.8	79.4 ± 3.4	95.9 ± 4.8	78.5 ± 5.4	94.7 ± 6.2
	CHO	83.3 ± 8.3	81.2 ± 8.1	84.7 ± 4.5	94.1 ± 5.5	81.0 ± 5.0	93.0 ± 4.1	77.0 ± 5.1	93.2 ± 6.0
	BIC	81.7 ± 7.5	81.1 ± 7.7	80.0 ± 5.9	89.6 ± 6.1	77.3 ± 8.2	90.2 ± 7.0	77.2 ± 6.9	91.6 ± 5.0

PCO_2_ (mmHg)	PASSIVE	38.5 ± 2.2	38.7 ± 2.0	35.8 ± 2.1	34.2 ± 5.4	28.0 ± 3.2	28.8 ± 2.5^*^	26.7 ± 2.3^*^	28.4 ± 2.6
	ACTIVE	37.9 ± 3.3	37.1 ± 3.5	36.3 ± 2.8	35.4 ± 4.7	32.2 ± 3.6	30.5 ± 3.6	32.0 ± 2.7	31.6 ± 2.9
	CHO	37.8 ± 1.9	37.5 ± 1.1	35.7 ± 3.4	33.8 ± 4.9	31.7 ± 2.6	32.0 ± 1.8	32.2 ± 2.7	31.9 ± 3.1
	BIC	39.0 ± 3.0	39.5 ± 3.7	38.5 ± 3.2	37.7 ± 5.9	33.3 ± 1.7^$^	33.4 ± 2.9	33.8 ± 3.0	32.4 ± 4.2^$^

^*^Different to all other interventions.

^†^Different to CHO.

^‡^Different to ACTIVE.

^$^Different to PASSIVE.
